# Parent Attributions of Ambiguous Symptoms in Their Children: A Preliminary Measure Validation in Parents of Children with Chronic Pain

**DOI:** 10.3390/children5060076

**Published:** 2018-06-13

**Authors:** Lauren C. Heathcote, Sara E. Williams, Allison M. Smith, Christine B. Sieberg, Laura E. Simons

**Affiliations:** 1 Department of Anesthesiology, Perioperative, and Pain Medicine, Stanford Medical School, Stanford, CA 94304, USA; lcheath@stanford.edu; 2 Division of Behavioral Medicine and Clinical Psychology, Cincinnati Children’s Hospital Medical Center and Department of Pediatrics, University of Cincinnati College of Medicine, Cincinnati, OH 45229, USA; Sara.Williams@cchmc.org; 3 Department of Pediatrics, University of Cincinnati College of Medicine, Cincinnati, OH 45267, USA; 4 Departments of Anesthesiology and Psychiatry, Boston Children’s Hospital, Harvard Medical School, Boston, MA 02115, USA; Allison.Smith@childrens.harvard.edu (A.M.S.); Christine.Sieberg@childrens.harvard.edu (C.B.S.)

**Keywords:** symptom attribution, Symptom Interpretation Questionnaire, somatic symptoms, pediatric chronic pain, parent, child

## Abstract

How parents attribute cause to their child’s physical symptoms is likely important in understanding how the parent responds to the child, as well as the child’s health outcomes, especially within the context of chronic illness. Here, we adapt the Symptom Interpretation Questionnaire for parent report (SIQ-PR) and provide preliminary validation in a sample of parents of children with chronic pain (*N* = 311). Confirmatory factor analysis revealed that the SIQ-PR structure is consistent with the original measure, with three distinct attribution types: psychological (emotional/affective), somatic (illness/disease), and environmental (situational/transient) causes. All three subscales demonstrated satisfactory to good internal consistency, and temporal stability. Parents typically endorsed more than one attribution for each symptom, indicating that parents of children with chronic pain have a multidimensional interpretation of physical symptoms in their children. Further, parent psychological and somatic attributions, but not environmental attributions, were significantly associated with (i) parent protective responses towards their child, and (ii) the child’s self-reported somatic and psychological symptoms, indicating convergent and divergent validity. The SIQ-PR may be a useful measure for future studies investigating intergenerational and interpersonal models of pediatric chronic pain, and more broadly, to examine parent attributions of children’s ambiguous symptoms within the context of childhood chronic illness.

## 1. Introduction

Physical symptoms, such as pain, a pounding heart, and breathlessness, are often ambiguous, and demand interpretation. Deciding what has caused these symptoms, our *symptom attributions,* is central to our experience of those symptoms [1,2], and predicts healthcare utilization [3]. One of the most widely used measures of symptom attribution is the Symptom Interpretation Questionnaire (SIQ) [3], which comprises three dimensions of symptom attributions: environmental (situational/transient causes), psychological (emotional/affective causes), and somatic (illness/disease causes). The SIQ was originally developed and validated as a self-report measure in a student sample [3], however, it has since been applied to clinical populations [4,5,6]. Studies with adults indicate that somatic and psychological attributions are associated with increased physical and emotional symptomatology and poor health outcomes [3,6,7,8]. Environmental attributions, on the other hand, are typically not associated with somatic or psychological symptomatology or illness [3], and have been considered more normative. Although context likely plays a role in specific symptom attributions, symptom attributions have been shown to be somewhat stable among individuals and over time [3].

Despite growing research in adults, little is known about symptom attributions among pediatric patients or their parents. Pediatric chronic pain is a context in which parent symptom attributions may be particularly relevant. Pediatric chronic pain patients often present with a myriad of physical [9] and psychological [10] symptoms, in tandem with their primary pain complaint. Moreover, symptom attributions may be passed down from parents to their children. Intergenerational [11] and interpersonal [12,13] models of chronic pain propose that parent beliefs about pain, including symptom attributions, likely drive parent behavior, as well as pain-specific social learning. Specifically, on the basis of these models, parent attributions of their child’s symptoms likely impact the parents’ responses to their child’s pain, as well as the child’s own pain and symptom experiences, and thus, may be critical in the etiopathogenesis of pediatric chronic pain disorders. Understanding parent attributions of child symptoms and how this relates to both parent and child outcomes can inform our understanding of familial factors within pediatric chronic pain.

To date, only two published studies have examined parent symptom attributions for children with chronic pain [14,15]. In both cases, findings revealed that parents typically attribute their child’s primary pain complaint to both somatic and psychological causes. While these studies examined youth with abdominal pain, parent symptom attribution is likely relevant across children with other pain complaints as well (e.g., musculoskeletal pain, headache, and neuropathic pain conditions) but has yet to have been investigated in a broader pain sample. Also, prior studies focused exclusively on pain-specific attributions. It is unknown how parents may discern other non-pain symptoms that their child may have or develop. In short, no studies have investigated broader (i.e., non-pain) symptom attributions in parents of children with chronic pain conditions, or how this relates to parent and child clinical outcomes. This may be, in part, due to the lack of available, well-validated measurement tools to assess parent attributions of child symptoms in general.

The current study provides preliminary validation of the SIQ adapted for parent report (SIQ-PR) in a sample of parents of children with various chronic pain conditions, by confirming the measure’s three-factor structure, and establishing adequate reliability (internal consistency and one-month stability). We also sought to provide preliminary evidence of convergent and divergent validity by examining the relative contribution of the three-symptom attribution factors/scales in explaining (i) parent protective responses towards their child, and (ii) the child’s somatic and psychological symptoms. Specifically, we predicted that parent psychological and somatic attributions (convergent), but not environmental attributions (divergent), would be associated with more protective behaviors towards the child, and greater child somatic and psychological symptoms.

## 2. Methods

### 2.1. Participants

Potential participants included parents of patients ages 8–18 years who underwent a multidisciplinary pain evaluation at a tertiary pain clinic in a large pediatric hospital in northeastern United States. Data were collected over a 21-month period. This sample was drawn from a larger study investigating fear-avoidance processes in pediatric chronic pain [16]. Of the 399 consecutive families approached to participate, 88 (22%) declined or were ineligible. Thus, the total sample included 311 patients and their parents, predominantly mothers (91%). Child patients were primarily White (White: 92%; Asian: 2%; Black: 1%; American Indian: 0.5%; other (biracial): 4.5%) and female (83.0%), reflective of the population of children seen in this tertiary care chronic pain clinic setting. The mean age of child patients was 13.75 years (standard deviation (SD) = 2.44). Family socioeconomic status (SES), based on the four-factor index of social status, ranged from 12 (semi-skilled workers) to 66 (business owner; professional), with a mean of 45.41 (SD = 12.37). The majority of mothers (58.3%) and fathers (54.2%) were college graduates. Children’s primary medical diagnoses included musculoskeletal pain (31.8%), neuropathic pain (32.5%; e.g., complex regional pain syndrome, neuralgia), headaches (2.3%; e.g., migraine, tension-type headache, combined and daily chronic headache), back/neck pain (13.8%; including scoliosis and idiopathic pain), abdominal pain (9.3%; e.g., functional abdominal pain), gynecological/genitourinary pain (5.8%), and other pain (3.5%; e.g., chest, ear). At the time of the evaluation, the patients’ median duration of pain was greater than one year (median = 14.6 months; range = 1–206 months). A small percentage (8%) of the sample reported experiencing pain for less than three months; however, as this was a treatment-seeking clinical sample, these participants were retained in analyses. This study was approved by Boston Children’s Hospital Institutional Review Board (ethical approval code: X08-09-0431; date of approval: 10 September 2008).

### 2.2. Measures

#### 2.2.1. Symptom Interpretation Questionnaire-Parent Report (SIQ-PR)

The SIQ-PR is an adapted version of the SIQ [3] used to assess causal attributions of commonly experienced physical symptoms. The original SIQ consists of 13 common physical symptoms, each followed by three items offering potential somatic (illness/disease), psychological (emotions/stress), and environmental (external/normalizing) causes (i.e., explanations). Respondents are required to rate the degree to which they would endorse each of the three causes for each symptom, on a four-point Likert-type scale (0 = not at all to 3 = a great deal). Thus, in total, respondents provide ratings for 39 attributions across the measure. Responses are summed by attribution subtype, yielding three subscale scores (somatic, psychological, environmental), with a possible range of 0–39 for each. Higher scores suggest stronger endorsement for each causal attribution subtype. The original SIQ is worded such that participants rate their own symptom attributions; the SIQ-PR was reworded so that parents rate their attributions should their child experience the symptoms. For example:

If my child had a prolonged headache, I would probably think that it is because:

1. He/she is emotionally upset.
2. There is something wrong with his/her muscles, nerves, or brain.
3. A loud noise, bright light, or something else irritated him/her.

See Table S1 in the supplementary materials for the complete measure and individual item means and standard deviations.

#### 2.2.2. Measures to Examine Convergent and Divergent Validity

##### Parent Protective Behaviors

The Adult Responses to Children’s Symptoms (ARCS) [17] assesses parent behavioral responses to children’s pain in three subscales; parent protectiveness, minimization of pain, and encouraging and monitoring. For the present study, the protectiveness subscale was used exclusively, given that it is most strongly and consistently associated with child outcomes as well as parents’ psychological flexibility and acceptance regarding their child’s pain [18]. The stem for each item is: “When your child has pain, how often do you…?”. Responses are rated on a five-point Likert-type scale (0 = never to 4 = always). The protectiveness subscale scores is computed by calculating the mean rating for item on the subscale; thus, the total possible range is 0–4. Higher scores indicate higher levels of parent protective behaviors.

##### Child Somatic Symptoms

The Child Somatization Inventory [19] (CSI) is a 35-item measure in which children self-report how much they were bothered by nonspecific physical symptoms (e.g., weakness, dizziness) in the previous two weeks, using a five-point Likert-type scale (0 = not at all to 4 = a whole lot). Total scores are computed by summing the items, with a possible range of 0–140. Higher scores indicate higher levels of somatic symptoms.

##### Child Psychological Symptoms

Two child self-report measures were used to assess children’s psychological symptoms: the Children’s Depression Inventory (CDI) [20] and the Multidimensional Anxiety Scale for Children (MASC) [21]. The CDI is a 27-item self-report measure of children’s depressive symptoms. Items are rated on a three-point scale (0–2) and are summed to obtain a total score with a possible range of 0–54. Higher scores indicate higher levels of depressive symptoms. The MASC is a 39-item self-report measure assessing four domains of anxiety in children: physical symptoms, social anxiety, separation anxiety, and harm avoidance. Items are rated on a four-point scale, from 0 to 3, and can be summed to obtain a total score with a possible range of 0–117. Higher scores indicate higher levels of anxiety symptoms. While T-scores are available for these measures, we used raw scores to permit the sensitivity analyses described below.

### 2.3. Procedure

Several study measures were collected as part of the standard clinical assessment battery, delivered in the context of the child’s multidisciplinary pain evaluation. Specifically, a demographics form, CSI, CDI, and ARCS were mailed to families prior to the evaluation, as part of standard clinic procedure. If parent or child questionnaires were incomplete upon arrival to their appointment, they were asked to do so prior to the start of the evaluation. The SIQ-PR and MASC were administered for research purposes in person on the day of the evaluation, as part of a larger institutional review board-approved research protocol [16]. Parents and children were approached by a research assistant prior to their evaluation with consent/assent obtained for the use of data from the clinical assessment battery in addition to study measures. To examine one-month stability of the SIQ-PR, parents were mailed a paper version of the SIQ-PR to complete, again, four weeks after their clinic visit. Of these parents, 199 (64% of the original sample) completed and returned the second questionnaire. There was a modest amount of missing data due to missed or omitted items. After requiring >85% of non-missing items across each subscale to qualify as a completed questionnaire, data from 190 (61%) parents were ultimately included in the final one-month stability analysis.

### 2.4. Statistical Analyses Plan

Data were analyzed with SPSS version 22.0 (IBM Corp. Armonk, NY, USA) and AMOS version 21 (IBM Corp.). For all analyses, *p* < 0.05 was the cut-off for statistical significance, but exact *p* values are reported in the text to aid critical interpretation of the data. Corrected values are reported for data that did not meet assumptions for equal variances. All variables were examined for skewness, kurtosis, and outliers prior to formal data analysis, and unless otherwise stated, all variables approximated a normal distribution.

#### 2.4.1. Psychometric Properties of the SIQ-PR 

We examined the psychometric properties of the SIQ-PR data in the chronic pain parent sample. Descriptive statistics were obtained to examine underlying assumptions of normality for all SIQ-PR items. Inter-item correlations (Cronbach’s α) were calculated for each scale to examine internal consistency. Pearson correlation coefficients were also calculated to investigate one-month stability. Next, confirmatory factor analysis (CFA) was conducted to confirm the pre-established three-factor structure of the original measure (somatic, psychological, environmental). Factor analytic guidelines recommend 5–10 subjects per item. With 39 items, we have approximately eight subjects per item for our analyses. Confirmatory factor analysis was conducted with AMOS version 21 (IBM Corp.). Confirmatory factor analysis is a type of structural equation modeling (SEM) that examines the fit between a predetermined factor structure, derived from exploratory factor analyses or prior research, and the sample data. A model was specified that included the items and corresponding factors based on the initial validation study of the SIQ [3]. Questionnaire items were treated as continuous variables in the CFA, consistent with the Likert-type response options. Full information maximum likelihood estimation (FIML) was employed. Based on recommendations by Ullman and Bentler [22], and Bentler and Bonett [23], the following statistics were used to evaluate model fit: *χ*^2^, *χ*^2^/degrees of freedom (*df*) (<2 acceptable), and root mean square error of approximation (RMSEA; <0.08 acceptable, <0.05 excellent).

#### 2.4.2. Associations Between SIQ-PR and Parent/Child Variables 

Pearson correlations (*r*) were performed to examine associations between parent symptom attributions and related parent and child constructs. Given that age was significantly correlated with child somatic symptoms (CSI: *r* = 0.14, *p* = 0.015), and that there were significant sex differences in child somatic symptoms (CSI: *t*(107.49) = 2.37, *p* = 0.019), we performed additional hierarchical regression analyses to control for these demographic factors. To be conservative, we performed these analyses for all child variables showing a significant association with parent symptom attributions, including age and sex, in a first step, and parent symptom attribution scale in a second step. 

#### 2.4.3. Sensitivity Analyses 

Given that measures of psychological symptoms (anxiety, depression) often include anxiety- or depression-linked physical symptoms (e.g., feeling tense or fatigued), we conducted sensitivity analyses with psychological symptom measures by removing physical items from these measures and re-running correlation analyses. This approach reflects a broader well-recognized issue [24,25,26] of using self-report depression (and anxiety) measures to assess psychological disorders in the context of chronic pain, wherein it is noted that reported elevated depressive levels in individuals with chronic pain may be partly explained by physical symptom item content on measures of depression that also characterizes chronic pain. For the CDI, we removed four items that reflect physical symptoms (sleep, fatigue, appetite, pain). For the MASC, we removed the physical symptoms subscale (12 items), thus computing total score from the remaining three subscales.

## 3. Results

### 3.1. Descriptive Statistics of the SIQ-PR

We first examined the means, standard deviations, skew, and kurtosis for all 39 individual items of the SIQ-PR. The mean scores (M) for the environmental attribution scale (M = 19.33, SD = 6.84, range = 3–37), the psychological attribution scale (M = 12.05, SD = 7.31, range = 0–37), and the somatic attribution scale (M = 10.14, SD = 5.58, range = 0–30), all approximated a normal distribution. As was the case for the original SIQ, the distribution of ratings for each of the three classes of causal attributions across the 13 symptoms suggested that, for most symptoms, each type of cause represented a plausible alternative explanation to the subjects. Four somatic attribution items were significantly positively skewed or kurtotic (>2) (mouth dry—salivary gland problem; heart pounding—heart problem; fatigue—anemia; hand shaking—neurological problem), indicating low item endorsement. Given that the remaining two attributions (i.e., psychological and environmental) for these particular symptoms were normally distributed, we chose to retain the skewed somatic items in analyses, in order to keep the measure equally representative of all three attributions types for all 13 items. The four skewed/kurtotic somatic items were examined carefully in the factor analysis to ensure they loaded adequately on the theorized factor. 

### 3.2. Confirming Factor Structure and Examining Reliability of the SIQ-PR

**Figure 1:**
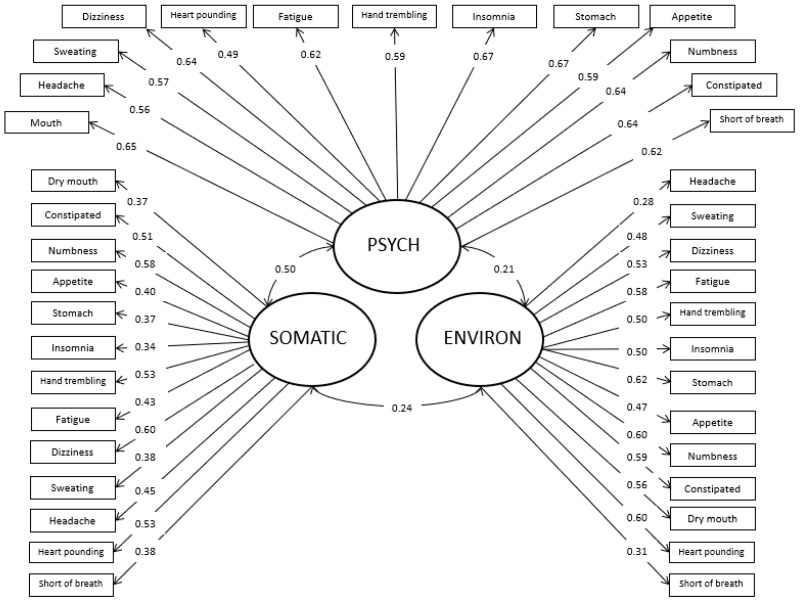
Confirmatory Factor Analysis model of Symptom Interpretation Questionnaire for parent report (SIQ-PR) with factor loadings.

We next performed a CFA to verify that the three-factor structure of the original SIQ (somatic, psychological, environmental) was retained with the parent version. The three-factor model was tested with structural equation modeling in order to verify the factor structure among parents of children with chronic pain, and examine overall model fit. The model provided good fit to the data [*χ*^2^(699) = 1352.49 *p* < 0.01; *χ*^2^/df = 1.94; RMSEA = 0.055 (90% confidence interval (CI) = 0.051–0.059)]. All factor loadings were significant, including the four somatic items with skewed distributions. See Figure 1 for the CFA model with factor loadings. In order to further assess the appropriateness of the current model, the proposed three-factor solution was compared to an alternative model with two-factors (environmental and somatic/psychological combined), given that the two latter dimensions are hypothesized to be more maladaptive than the former. The two-factor model resulted in a significant deterioration in model fit when compared to the three-factor model (Δ*χ*^2^(1) = 285.37, *p* < 0.001), indicating that the three-factor solution provided a significantly better fit to the data.

All three scales exhibited satisfactory to good reliability with Cronbach’s α of 0.88 for the psychological subscale, 0.76 for the somatic subscale, and 0.82 for the environmental subscale. One-month stability estimates were also satisfactory, with Pearson *r* correlations of 0.71 (*p* < 0.001) for the psychological subscale, 0.66 (*p* < 0.001) for the somatic subscale, and 0.61 (*p* < 0.001) for the environmental subscale.

### 3.3. Associations between Parent Symptom Attributions and Parent and Child Variables

We performed bivariate Pearson correlations between the three SIQ-PR subscale scores and parent protective behaviors, child somatic symptoms, and child psychological symptoms (depression, anxiety). All correlations are displayed in Table 1. We also present descriptive statistics (M, SD) for parent and child self-report measures to aid interpretation of the data.

#### 3.3.1. Parent Variables 

As expected, parent attributions of child symptoms were associated with other measures of the parent’s behavioral responses to their child’s pain. Higher parent endorsement of somatic and psychological symptom attributions was significantly, though modestly associated with more protective behaviors in response to their child’s pain (range: *r* = 0.22 to 0.23). Environmental symptom attributions were not significantly associated with parent protective behaviors.

#### 3.3.2. Child Variables

As expected, parent attributions of child symptoms were associated with the child’s reporting of their own somatic and psychological symptoms. In particular, higher parent endorsement of psychological symptom attributions was significantly associated with all three child measures: child somatic symptoms, child anxiety symptoms, and child depressive symptoms. However, higher parent endorsement of somatic symptom attributions was significantly associated with the child’s somatic and anxiety symptoms, but not with the child’s depressive symptoms. Again, parent environmental symptom attributions were not associated with any child-reported measures. Where significant, Pearson correlations were small to medium in effect size (range: *r* = 0.16 to 0.35), with the strongest associations between parent psychological attributions and child psychological symptoms (anxiety and depressive symptoms). Follow-up regression analyses controlling for demographic factors (child age and sex) confirmed that parent symptom attributions remained significant predictors of child symptoms across all analyses (see Table S2).

### 3.4. Sensitivity Analyses

We conducted sensitivity analyses to confirm if the above correlations remained significant when removing somatic items from child measures of anxiety and depression (see statistical analysis plan). Findings did not change for either child depressive or anxiety symptoms, indicating that the removal of somatic items from psychological measures did not impact findings. Overall, correlation coefficients were slightly reduced for all sensitivity analyses (see Table 2).

## 4. Discussion

In this study, we adapted the SIQ [3] for parent report, providing preliminary examination of its psychometric properties in a large sample of parents of children with chronic pain. We confirmed the measure’s original three-factor structure, representing three attribution types: psychological (emotional/affective causes), somatic (illness/disease causes), and environmental (situational/transient causes). In line with previous studies using the original measure, environmental attributions were most common, followed by psychological attributions, and then somatic attributions. All three subscales demonstrated satisfactory to good internal consistency and temporal stability. Further, parent psychological and somatic attributions of their child’s symptoms, but not environmental attributions, were significantly associated with (i) parent protective responses towards their child, and (ii) the child’s somatic and psychological symptoms. Of note, associations were strongest between psychological attributions and psychological symptoms, and between somatic attributions and somatic symptoms. Taken together, preliminary evidence is provided for acceptable reliability, as well as convergent and divergent validity between the three subscales.

This study contributes to the limited existing literature on parent symptom attribution within the context of chronic pain. While earlier studies focused on maternal attributions of children’s primary (abdominal) pain complaint [14,15], this is the first study to consider how parents attribute cause to other physical symptoms that are often comorbidly experienced by young people with chronic pain, in a broader sample of patients with chronic pain. One notable finding is that parents typically endorsed more than one cause for each symptom. These findings are in line with the study by Claar and Walker [14], suggesting that parents typically have a multidimensional, rather than a dualistic or threat-focused attributional style of interpreting physical symptoms in their children. An interesting next step would be to examine how attributions differ between parents of children with chronic pain compared to parents of children with or without other chronic illnesses. Adult findings in this area are mixed: some studies find that clinical groups endorse fewer environmental attributions than healthy controls [3,27], while others find no differences in environmental attributions between patient and non-patient groups [28]. Also relevant will be an understanding of how parent attribution of child symptoms impacts parent reporting of those symptoms within the context of pain assessment, for example, in a clinical setting. 

This study also adds to a growing understanding of intergenerational and interpersonal factors associated with pediatric chronic pain. A number of recent theoretical models [11,29] propose that the way(s) in which parents respond to their own and their child’s symptoms (including pain) likely influences children’s development of health attitudes and behaviors that, in maladaptive instances, may overemphasize physical symptoms and reinforce children’s attention to these symptoms. Our findings provide initial evidence that the way(s) in which parents attribute cause to their child’s symptoms is indeed associated with their (protective) behaviors towards the child. This is in line with previous studies showing that parent cognitions (e.g., catastrophizing about their child’s pain) are associated with protective behaviors towards the child [30,31]. These protective behaviors, in turn, have been shown in previous studies to contribute to children’s maladaptive pain outcomes [32,33,34]. Here, we also find that parent attributions of child symptoms are associated with the child’s own symptomatology. Given these findings, a useful next step would be to investigate if parent protective behaviors mediate the association between parent symptom attributions and child symptoms. If experimental or longitudinal studies confirm that parent symptom attributions indeed causally contribute to parent protective behaviors, then parent symptom attributions may be a potential target for intervention.

Another outstanding question is whether the impact of parent symptom attributions differ according to children’s age and developmental stage. Indeed, we included a wide age range of child participants (8–18 years). Follow-up regression analyses controlling for demographic factors (child age and sex) confirmed that parent symptom attributions remained significant predictors of child symptoms across all analyses (see Table S2); however, studies with equally well-powered samples across smaller age ranges will be useful to disentangle potential age effects. Another useful next step to test social learning mechanisms [11] will be to understand how parent attributions of their child’s symptoms relate to (i) parent attributions of their own symptoms, and (ii) children’s attributions of their own symptoms. Parents’ own general symptom attributions may be a lens through which they view their child’s symptoms; however, this remains to be tested. Future studies using the SIQ-PR should consider measuring the parents’ own medical illness or psychiatric history to best understand whether and how parents’ previous experiences influence symptom attributions in their children. Daily diary studies, in which the parent and child complete daily ratings of attributions, behaviors, and symptoms (e.g., [35]), would be particularly useful for examining causal relationships. In addition to age, future studies should also recruit more ethnically diverse samples, in order to consider how parent cultural values are relevant to their interpretation of physical symptoms in their children. 

One consistent finding throughout this study is that parents’ environmental attributions were not associated with parent or child factors. These findings are in line with expectations and with adult studies showing that these normalizing attributions are infrequently linked with negative health outcomes [3]. An outstanding question is whether or not these attributions are protective of such maladaptive outcomes. We found no evidence in this study that environmental attributions were negatively associated with children’s somatic or psychological symptomatology, which does not support the idea that such attributions are protective per se. A related question is how parent symptom attributions contribute to health-care utilization in youth with chronic pain. Indeed, young people with chronic pain often report frequent medical encounters that are typically initiated by the parent. Parent attributions of their child’s symptoms may be one factor driving such help-seeking behaviors. A previous study indeed indicated that environmental symptom attributions contributed to non-help-seeking behaviors in adults with fibromyalgia [36]. If we consider somatic (illness) attributions as threat-focused attributions, then these findings could be considered within the broader framework of threat perception within the context of chronic pain [37]. Previous studies with adults [38], and more recently, with young people [39], indicate that individuals with chronic pain exhibit a negative, threat-focused interpretation bias when appraising bodily sensations and health. Regardless, recognizing that there are biopsychosocial contributors to pain, and thus, encouraging a multidimensional attribution pattern, rather than simply discouraging somatic attributions, may be most beneficial in this population.

This study has limitations, indicating directions for future research. First, the majority of parent participants in this study were mothers; thus, it is unclear to what extent paternal symptom attributions play a similar role. Second, the majority of children reported musculoskeletal pain, with fewer reporting abdominal pain or headaches. While musculoskeletal pains are most commonly reported in adolescent samples [40], future studies, especially with younger samples, should examine whether these findings extend to non-musculoskeletal pain complaints. The SIQ-PR may also be a useful measure for investigating the impact of parent symptom attributions in the context of other childhood chronic illnesses, where symptom burden is high, such as cystic fibrosis, asthma, and cancer. Third, we included a treatment-seeking sample in which parents may be less likely to endorse environmental attributions of their child’s symptoms; thus, future studies should also recruit families from the community. Fourth, the SIQ-PR measures parent attributions for potential symptoms in their child, not their child’s actual symptoms. There are benefits of using such a general measure; responses are standardized across respondents, and results point to more general attributions that have implications for parent attributions for new symptoms that their child may develop. Additionally, our findings from the child measures (CSI, MASC, CDI) confirmed that the young patients included in this study do indeed experience physical symptoms beyond their primary pain complaint. However, a full understanding of the role and nature of parent symptom attributions will include complimentary investigations of how parents attribute causes to actual symptoms that their child experiences. Indeed, parent attributions of their child’s symptoms in everyday life likely emerge as complex, dynamic cognitions that change in response to the child’s experience of those symptoms and related distress over time. Future studies may also consider adding a forced choice component, in which parents are asked to select the attribution that they find most convincing, rather than rating all attribution types for each item. Fifth, attributions of the cause of physical symptoms is just one component of how a caregiver represents their child’s illness. Illness representations more broadly also include time course, consequences, and curability of the condition. These other dimensions have also been shown to explain help-seeking behaviors, compliance with treatment regimens, and success in coping with chronic illness (see [3] for summary). Indeed, associations between symptom attributions and child and parent factors reported in the current study were mostly small to medium. A good understanding of how children with chronic pain experience physical symptoms, including healthcare utilization and clinical outcomes, will likely necessitate an integration of all of these factors.

There is increasing evidence that the beliefs and behavior of primary caregivers play a crucial role in children’s experiences of chronic illness. In this study, we adapted the SIQ for parent report, providing a new measure that can be used to examine how parents attribute cause to their child’s physical symptoms within the context of chronic illness. We provide preliminary evidence that parent attributions of child symptoms within the context of child chronic pain is associated with parent behavioral responses towards the child, and the child’s self-reported symptoms. Findings indicate that parent symptom attributions may be one important factor to consider when examining intergenerational and interpersonal factors that contribute to the experience of chronic illness in youth.

## Figures and Tables

**Table 1:** Pearson correlation matrix.

Variable	1	2	3	4	5	6	7	Mean	SD
SIQ-PR subscales									
1. Environmental attributions	-	0.210 ***	0.245 ***	−0.063	−0.098	0.000	−0.023	19.3	6.8
2. Psychological attributions		-	0.428 ***	0.222 ***	0.249 ***	0.345 ***	0.330 ***	12.0	7.3
3. Somatic attributions			-	0.231 ***	0.188 **	0.155 **	0.073	10.1	5.6
Parent									
4. Protective behaviors				-	0.328 ***	0.254 ***	0.263 ***	1.4	0.6
Child									
5. Somatic symptoms					-	0.405 ***	0.483 ***	30.5	17.8
6. Anxiety symptoms						-	0.521 ***	42.9	19.1
7. Depressive symptoms							-	10.0	7.2

** *p* < 0.01, *** *p* < 0.001; shading intensity represents strength of association. SD, standard deviation.

**Table 2:** Comparison of correlations with main and sensitivity analyses.

	Child Anxiety Symptoms	SA: Child Anxiety Symptoms	Child Depression Symptoms	SA: Child Depression Symptoms
Environmental attributions	0.00	−0.00	−0.02	−0.02
Psychological attribution	0.35 ***	0.26 ***	0.33 ***	0.27 ***
Somatic attributions	0.16 **	0.12 *	0.07	0.03

* *p* < 0.05, ** *p* < 0.01, *** *p* < 0.001; SA = Sensitivity analysis.

## Supplementary Materials

The following are available online at http://www.mdpi.com/2227-9067/5/6/76/s1, Table S1: SIQ-PR item means and standard deviations, Table S2: Hierarchical stepwise regressions to confirm significant associations between parent attributions and child variables whilst controlling for child age and sex.
