# Supplementary material for: Parent Attributions of Ambiguous Symptoms in Their Children: A Preliminary Measure Validation in Parents of Children with Chronic Pain
Source: Children (Basel). 2018 Jun 13;5(6):76. doi: 10.3390/children5060076 (PMC6025587; doi:10.3390/children5060076)
Supplement: Supplementary file 1 [file children-05-00076-s001.pdf]

Supplementary Materials

Table S1. SIQ-PR item means and standard deviations.

Item	M (SD)
**1. If my child had a *prolonged headache,* I would probably think that it is because:**	
He/she is emotionally upset	0.84 (0.81)
There is something wrong with his/her muscles, nerves, or brain	0.94 (1.02)
A loud noise, bright light or something else irritated him/her	0.82 (0.93)
**2. If my child was *sweating a lot*…**	
He/she must have a fever or infection	1.06 (1.05)
He/she is anxious or nervous	0.72 (0.84)
The room is too warm, he/she is overdressed or working too hard	1.84 (0.95)
**3. If my child got *dizzy all of a sudden*…**	
There is something wrong with his/her heart or blood pressure	0.60 (0.91)
He/she is not eating enough or got up too quickly	1.85 (0.89)
He/she must be under a lot of stress	0.77 (0.88)
**4. If my child noticed his/her *mouth was dry*…**	
He/she must be scared or anxious about something	0.46 (0.73)
He/she needs to drink more liquids	2.33 (0.77)
There is something wrong with his/her salivary glands	0.06 (0.24)
**5. If my child felt his/her *heart pounding*…**	
He/she has exerted him/herself or drunk a lot of caffeinated beverages	1.63 (1.05)
He/she must be really excited or afraid	1.65 (0.91)
There must be something wrong with his/her heart	0.25 (0.61)
**6. If my child felt *fatigued*…**	
He/she is emotionally exhausted or discouraged	1.31 (1.01)
He/she has been over-exerting him/herself or not exercising enough	1.65 (0.99)
He/she is anemic or his/her blood is weak	0.25 (0.57)
**7. If my child noticed his/her *hand trembling*…**	
He/she might have some sort of neurological problem	0.49 (0.84)
He/she is very nervous	1.27 (0.97)
He/she has tired the muscle in his/her hand	1.11 (1.01)
**8. If my child had *trouble sleeping*…**	
Some kind of pain or physical discomfort is keeping him/her awake	1.85 (0.99)
He/she is not tired or had too much caffeine	0.99 (0.96)
He/she is worrying too much or must be nervous about something	1.51 (0.93)
**9. If my child's *stomach was upset*…**	
He/she has worried him/herself sick	0.84 (0.91)
He/she has the flu or stomach irritation	1.48 (0.87)
He/she has had something to eat that did not agree with him/her	1.72 (0.87)
**10. If my child had *lost his/her appetite*…**	
He/she has been eating too much or his/her body doesn't need as much food as before	0.91 (0.98)
He/she is worrying so much that food just doesn't taste good anymore	0.58 (0.79)
He/she has some stomach or intestinal problem	1.13 (1.01)
**11. If my child had a *hard time catching his/her breath*…**	
His/her lungs are congesting from infection or irritation	0.78 (0.93)
The room is stuffy or there is too	0.75 (0.85)
He/she is over-excited or anxious	1.01 (0.93)
**12. If my child noticed *numbness or tingling in his/her hands or feet*…**	
He/she is under emotional stress	0.46 (0.77)
There is something wrong with his/her blood circulation	0.82 (0.91)
He/she is cold or his/her hand went to sleep	1.70 (0.99)
**13. If my child was *constipated or irregular*…**	
There is not enough fruit or fiber in his/her diet	2.09 (0.90)
Nervous tension is keeping him/her from being regular	0.70 (0.84)

Table S2. Hierarchical stepwise regressions to confirm significant associations between parent attributions and child variables whilst controlling for child age and sex.

Variable	B	SE B	β	R^2 Change
Outcome: Child somatic symptoms				
Step 1				
Child age	0.96	0.41	0.13*	0.03*
Child sex	−4.43	2.67	−0.09	
Step 2				
Child age	1.01	0.41	0.14*	0.04**
Child sex	−4.61	2.62	−0.10	
Parent somatic attributions	0.62	0.18	0.20**	
Outcome: Child somatic symptoms				
Step 1				
Child age	0.96	0.41	0.13*	0.03*
Child sex	−4.43	2.67	−0.09	
Step 2				
Child age	1.04	0.40	0.14*	0.06***
Child sex	−3.74	2.59	−0.08	
Parent psychological attributions	0.61	0.13	0.25***	
Outcome: Child anxiety symptoms				
Step 1				
Child age	−0.91	0.44	−0.12*	0.02*
Child sex	−5.17	2.88	−0.10	
Step 2				
Child age	−0.89	0.44	−0.11*	0.02**
Child sex	−5.22	2.85	−0.10	
Parent somatic attributions	0.53	0.20	0.15**	
Outcome: Child anxiety symptoms				
Step 1				
Child age	−0.91	0.44	−0.12*	0.02*
Child sex	−5.17	2.88	−0.10	
Step 2				
Child age	−0.80	0.42	−0.10	0.11***
Child sex	−4.24	2.72	−0.08	
Parent psychological attributions	0.88	0.14	0.34***	
Outcome: Child depression symptoms				
Step 1				
Child age	0.28	0.17	0.10	0.01
Child sex	−1.01	1.09	−0.05	
Step 2				
Child age	0.32	0.16	0.11	0.11***
Child sex	−0.66	1.03	−0.04	
Parent psychological attributions	0.32	0.05	0.33***	

Note. *$p < 0.05$; **$p < 0.01$; *** $p < 0.001$
